# Burden of rotavirus and other microorganisms in hospitalized children with acute gastroenteritis in Yangon, Myanmar, before the introduction of rotavirus vaccine

**DOI:** 10.1016/j.ijregi.2025.100589

**Published:** 2025-01-31

**Authors:** Tatsuki Ikuse, Yuta Aizawa, Kazuhiro Kamata, Khin Nyo Thein, Di Ja Lasham, Su Sandar Tun, Nay Chi Win, Su Mon Kyaw Win, Ai Ito, Mon Mon, Aye Thida, Aye Aye Khin, Yuki Higashimoto, Tetsushi Yoshikawa, Satoshi Komoto, Hisami Watanabe, Reiko Saito, Akihiko Saitoh

**Affiliations:** 1 Department of Pediatrics, Niigata University Graduate School of Medical and Dental Sciences, Niigata, Japan; 2 Infectious Diseases Research Centre of Niigata University in Myanmar, Yangon, Myanmar; 3 Yankin Children Hospital, Yangon, Myanmar; 4 Department of Microbiology, University of Medicine 2, Yangon, Myanmar; 5 Department of Medical Education, University of Medicine 2, Yangon, Myanmar; 6 Rector, University of Medicine 2, Yangon, Myanmar; 7 Faculty of Cellular and Molecular Biology, Fujita Health University School of Medical Sciences, Toyoake, Japan; 8 Department of Pediatrics, Fujita Health University School of Medicine, Toyoake, Japan; 9 Department of Virology, Fujita Health University School of Medicine, Toyoake, Japan; 10 Division of International Health, Graduate School of Medical and Dental Science, Niigata University, Niigata, Japan

**Keywords:** Myanmar, Rotavirus, Acute gastroenteritis, Children, PCR

## Abstract

**Objectives:**

In developing countries, acute gastroenteritis (AGE) is a leading cause of death in children younger than 5 years. In Myanmar, no comprehensive study has been done to investigate the microorganisms responsible for AGE among hospitalized children. Multiplex polymerase chain reaction (PCR) was used to identify the microorganisms responsible for AGE in children hospitalized in Myanmar before the introduction of the rotavirus vaccine.

**Methods:**

This prospective study enrolled children younger than 12 years with AGE who were hospitalized at the Yankin Children's Hospital in Yangon, Myanmar, between September 2019 and February 2020. Multiplex PCR (FilmArray^TM^ GI panel, BioFire Diagnostics, Salt Lake City, USA) and genotyping with Sanger sequencing of rotavirus were performed. Clinical data, including disease severity, were collected from the medical records.

**Results:**

We collected stool samples from 92 patients. Multiple microorganisms (median 3; interquartile range 2-4) were detected in 81 patients (88%). Rotavirus and norovirus were detected in 77 (84%) and 33 patients (36%), respectively. The most frequent bacterial pathogen detected was Enteroaggregative *E. coli* (n = 62/92, 67%). The most common rotavirus genotypes were G1P [8] (19/73; 26%) and G2P [4] (19/73; 26%).

**Conclusions:**

Rotavirus is the predominant pathogen associated with AGE in hospitalized children in Myanmar. The introduction of a rotavirus vaccine will reduce the morbidity and mortality of children with rotavirus-associated AGE in Myanmar.

## Highlights

• Multiplex polymerase chain reaction was useful for detecting microorganisms of acute gastroenteritis.
• Multiple microorganisms were detected from the stool samples by multiplex polymerase chain reaction.
• Rotavirus was the dominant microorganism in Myanmar in the pre-rotavirus vaccine era.
• The most frequently detected rotavirus genotypes were G1P [8] and G2P [4].

## Introduction

Acute gastroenteritis (AGE) is a leading cause of death worldwide in children younger than 5 years, especially in low/middle-income countries, where poor hygiene and malnutrition are more prevalent [1]. Approximately 525,000 children die of diarrhea every year [2]. Reducing the incidence of AGE in children is therefore an important goal of the World Health Organization (WHO) [1].

Acute diarrhea is usually caused by AGE and is attributable to viral, bacterial, or parasitic infections [1]. Rotavirus is the most common pathogen detected in children younger than 5 years of age with severe AGE [2]. A global rotavirus surveillance study in 2013, which also examined regions without rotavirus vaccination, yielded a rotavirus detection rate of approximately 40% for all AGE cases among children younger than 5 years [2]. The remaining 60% of the cases are attributable to other viruses (such as norovirus, adenovirus, and astrovirus), bacteria (Campylobacter, enteropathogenic *Escherichia coli*, Salmonella, and Shigella), and parasites [3]. The WHO recommended global rotavirus vaccination after the introduction of rotavirus vaccines. Subsequently, a decrease in AGE due to rotavirus was observed in countries where routine rotavirus vaccines were introduced [4].

In Myanmar, a more than 60% reduction in mortality in children younger than 5 years was observed from 1990 to 2019 [5]. One contributing factor was the reduction in vaccine-preventable diseases (VPDs) by the Expanded Program on Immunization (EPI), which did not include rotavirus vaccines until February 2020 [5]. However, the mortality rate in this age group was 44.7 deaths per 1000 live births in 2019, and diarrhea was the leading cause of childhood death in Myanmar [6]. Therefore, the reduction of AGE-related mortality is a top priority. A previous WHO study in Myanmar, before the rotavirus vaccine introduction, reported that rapid antigen testing revealed rotavirus in almost half of the stools collected from children with AGE [6,7]. However, the details of non-rotavirus pathogens and the co-detection rates of microorganisms in stool samples have never been investigated in Myanmar.

Baseline data on the incidence of rotavirus and non-rotavirus infections in children with severe AGE in Myanmar before the introduction of the rotavirus vaccine are lacking. To analyze this baseline, this prospective study investigated the detection of microorganisms using multiplex polymerase chain reaction (PCR) in hospitalized children with AGE in Myanmar.

## Methods

### Study design and population

This prospective study investigated children younger than 12 years who were admitted to Yankin Children's Hospital (YKCH) in Yangon, Myanmar, with a diagnosis of AGE during the period from September 2019 through February 2020. This institution was selected as the study site as it is the largest children's hospital in Yangon, Myanmar. In addition, we previously conducted a clinical study related to severe acute respiratory infection in children at this institution [8]; thus, we had a close relationship and understanding with hospital staff members. This time period was selected because it encompasses both the Burmese rainy season (June to October) and dry season (November to February) and because it immediately preceded the introduction of rotavirus vaccination in Myanmar. Yankin Children's Hospital has 550 beds for children aged 1 month to 12 years. An AGE diagnosis had to satisfy all the following criteria: history of diarrhea, onset during the previous 7 days, and hospitalization due to poor oral intake, malnutrition, and/or dehydration. Patients with immunodeficiency were excluded from the study. Written informed consent was obtained from patients and their caregivers. This study was approved by the ethics committees of the Department of Medical Research, Ministry of Health and Sports, Myanmar (Ethics/DMR/2019/060E/2020) and Niigata University, Japan (2018-0428).

### Clinical data

We collected clinical data from the medical records of patients with AGE, including age, sex, date of onset of symptoms, hospitalization and discharge, vital signs, clinical symptoms, signs, treatment, outcome, and Vesikari score, which indicates AGE severity and comprises duration of vomiting and diarrhea, number of vomiting episodes and defecations, body temperature, severity of dehydration, and treatment [9]. Moderate-to-severe AGE was defined as a Vesikari score of 7-10, while a score of 11 or higher indicated severe AGE. Data for the variables used to calculate the Vesikari scores were collected from the patients’ medical charts, and the calculated scores were recorded in the chart.

### Sample collection and detection of microorganisms responsible for AGE

Stool samples were collected upon hospital admission of patients with AGE. Stool samples were suspended in 2 ml of buffer using FecalSwab^TM^ (Copan Italia s. p. a., Italy). Rapid antigen testing for rotavirus and adenovirus (Quick Chaser^TM^ Rota/Adeno, MIZUHO MEDY Co., Ltd., Japan) and light microscopic observation of parasite eggs were performed at YKCH. The remaining samples were stored at −80°C and sent to the laboratory of Niigata University. At Niigata University, multiplex PCR was performed by using the FilmArray^TM^ gastrointestinal (GI) panel (BioFire Diagnostics, Salt Lake City, UT) for all samples [10]. The FilmArray^TM^ GI panel targets Campylobacter (*Campylobacter jejuni, coli*, and *upsaliensis*), *Clostridioides difficile* (Toxin A/B), *Plesiomonas shigelloides*, Salmonella spp., *Yersinia enterocolitica*, Vibrio (*Vibrio parahaemolyticus, vulnificus*, and *cholerae*), *Vibrio cholera*, enteroaggregative *E. coli* (EAEC), enteropathogenic *E. coli* (EPEC), enterotoxigenic *E. coli* (ETEC), Shiga-like toxin-producing *E. coli* (STEC), *E. coli* O157, Shigella/enteroinvasive *E. coli* (EIEC), Cryptosporidium spp., *Cyclospora cayetanensis, Entamoeba histolytica, Giardia lamblia*, adenovirus F40/41, astrovirus, norovirus GI/GII, rotavirus A, and sapovirus (I, II, IV, and V).

### Rotavirus genotyping

Viral ribonucleic acid (RNA) was extracted from 140 μl of 10% stool suspensions by using a QIAamp Viral RNA Mini Kit (Qiagen, Hilden, Germany) in accordance with the manufacturer's instructions. To determine the G and P genotypes, reverse transcription followed by polymerase chain reaction was performed using the SuperScriptTM III One-Step RT-PCR System with a Platinum Taq High-Fidelity DNA Polymerase Kit (Invitrogen, Carlsbad, CA, USA). Primers were designed to amplify the common sequences of group A RV strains [[11], [12], [13]]. A BigDye Terminator V3.1 Cycle Sequencing Reaction Kit (Applied Biosystems, Foster City, CA, USA) was used for the Sanger sequencing of each PCR product. Sequencing products were analyzed using an ABI 3500 Genetic Analyzer (Applied Biosystems). Finally, G and P genotyping of the obtained sequences was performed using the Virus Pathogen Database and Analysis Resource (ViPR) tool (https://www.viprbrc.org/brc/rvaGenotyper?method=ShowCleanInputPage&decorator=reo).

### Statistical analysis

Descriptive statistics were reported as medians with interquartile ranges or percentages, as appropriate. Using the Mann-Whitney U test, we compared AGE severity (Vesikari score) in relation to rotavirus infection status and the number of pathogens (single vs multiple). All statistical analyses were performed using the STATA software version 16.1 (Stata Corp., College Station, TX, USA). A *P*-value of <0.05 was considered to indicate statistical significance.

## Results

### AGE cases in Yangon, Myanmar, 2019-2020

During the study period, stool samples were collected from 92 children admitted to the hospital with AGE (Table 1). The median patient age was 12.5 months (interquartile range [IQR], 8-18 months), and 39% (36/92) were male patients. The median Vesikari score was 11 (IQR: 9-13); 53% (49/92) had severe AGE (score: greater than 10), and 46% (42/92) had moderate-to-severe AGE (score: 7-10). No deaths occurred.

**Table 1:** Characteristics of AGE in children in Yangon, Myanmar.

Patient characteristics	N
Median age, months (IQR)	12.5 (8-18)
Sex (male), n (%)	36 (39%)
Month and year of admission^a^	
September 2019	4 (4.3%)
October 2019	2 (2.2%)
November 2019	27 (29.3%)
December 2019	25 (27.2%)
January 2020	17 (18.5%)
February 2020	17 (18.5%)
Vesikari Score	
Median score (IQR)	11 (9-13)
Mild (<7)	1 (1.1%)
Moderate to severe (7-10)	42 (45.7%)
Severe (>10)	49 (53.3%)
Duration of diarrhea, days (IQR)	1 (1-2)
Number of defecations per day, n (IQR)	3 (2-6)
Duration of vomiting, days (IQR)	1 (1-2)
Number of vomiting episodes, n (IQR)	2 (1-3)
Body temperature, (IQR)	38.0 (37.4-38.5)
Length of hospital stay, days (IQR)	3 (2-4)

IQR, interquartile range.

a In Myanmar, the rainy season is June-October, and the dry season is November-February.

### AGE microorganisms

**Figure 1:**
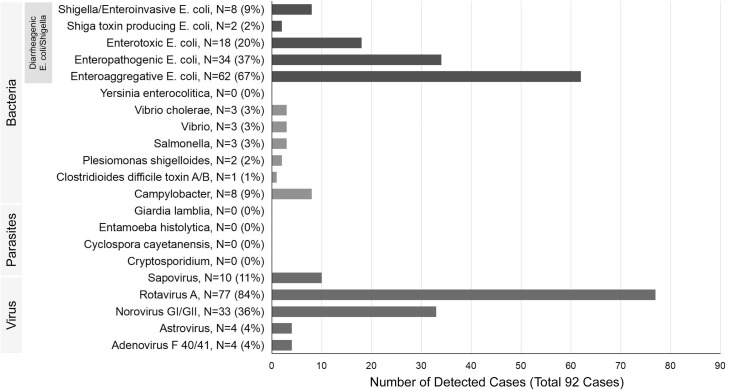
Details of microorganisms detected by the FilmArray^TM^ GI Panel in an analysis of stool samples from hospitalized children with acute gastroenteritis. Fifteen microorganisms were detected in diarrheal stools from children in Yangon, Myanmar, with acute gastroenteritis; rotavirus (77/92, 84%) was the most frequently detected microorganism.

**Figure 2:**
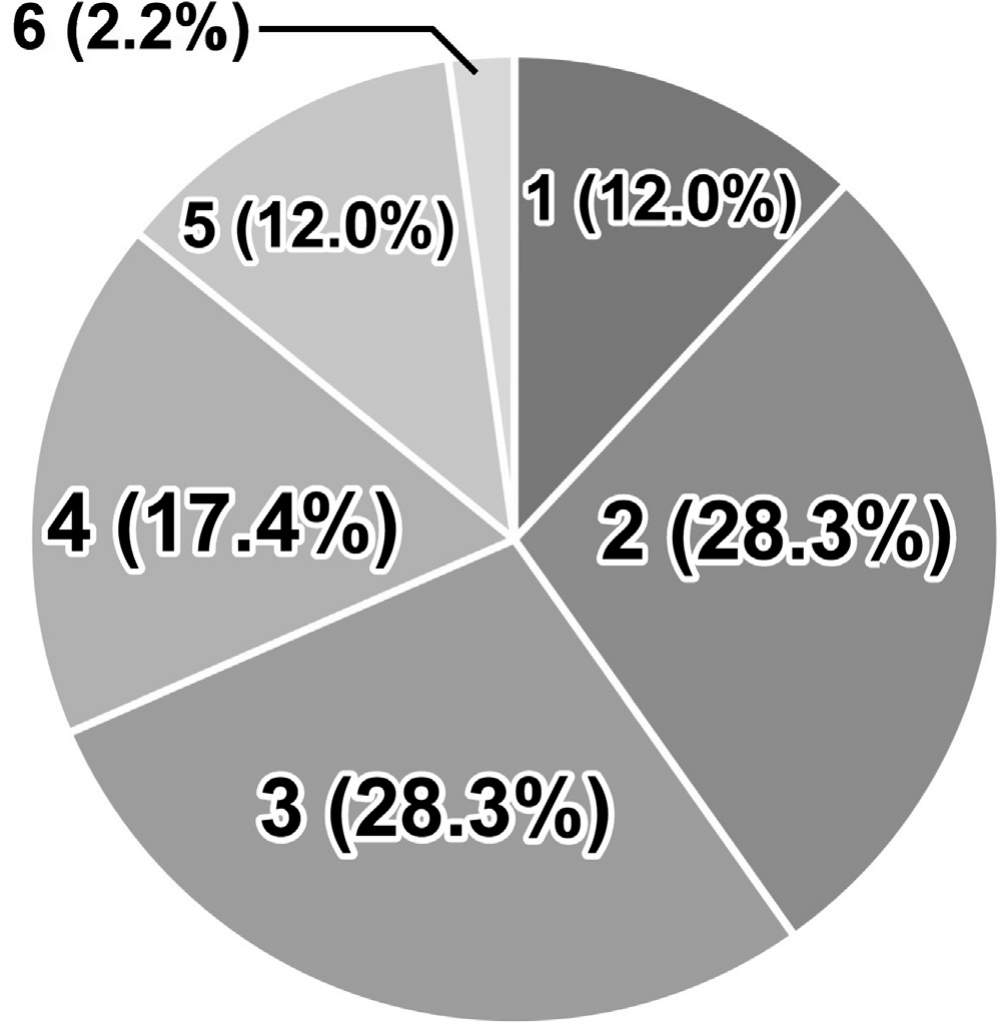
Number of microorganisms detected in stool samples. In total, 88% of children with acute gastroenteritis in Yangon, Myanmar had two or more microorganisms in their stools.

Among the 92 AGE cases, rapid antigen testing yielded positive results for rotavirus in 46/92 (50%) patients and for adenovirus in 2/92 (2%) patients. Multiplex PCR detected more than one of the 15 types of microorganisms in samples from 92 patients (100%). Rotavirus was the most frequently detected pathogen (77 patients; 84%) (Figure 1). Norovirus was the second most frequent viral pathogen (33/92, 36%), and EAEC, the most frequent bacterial pathogen, was detected in 62 patients (67%). Parasites were not detected. Most patients (88%) were infected with multiple microorganisms, and the median number of positive microorganisms in the stool was 3 (IQR 2-4) (Figure 2).

With multiplex PCR results as the standard, the sensitivity and specificity of the rapid viral antigen test were 53% and 67%, respectively.

### AGE severity by rotavirus infection status

**Figure 3:**
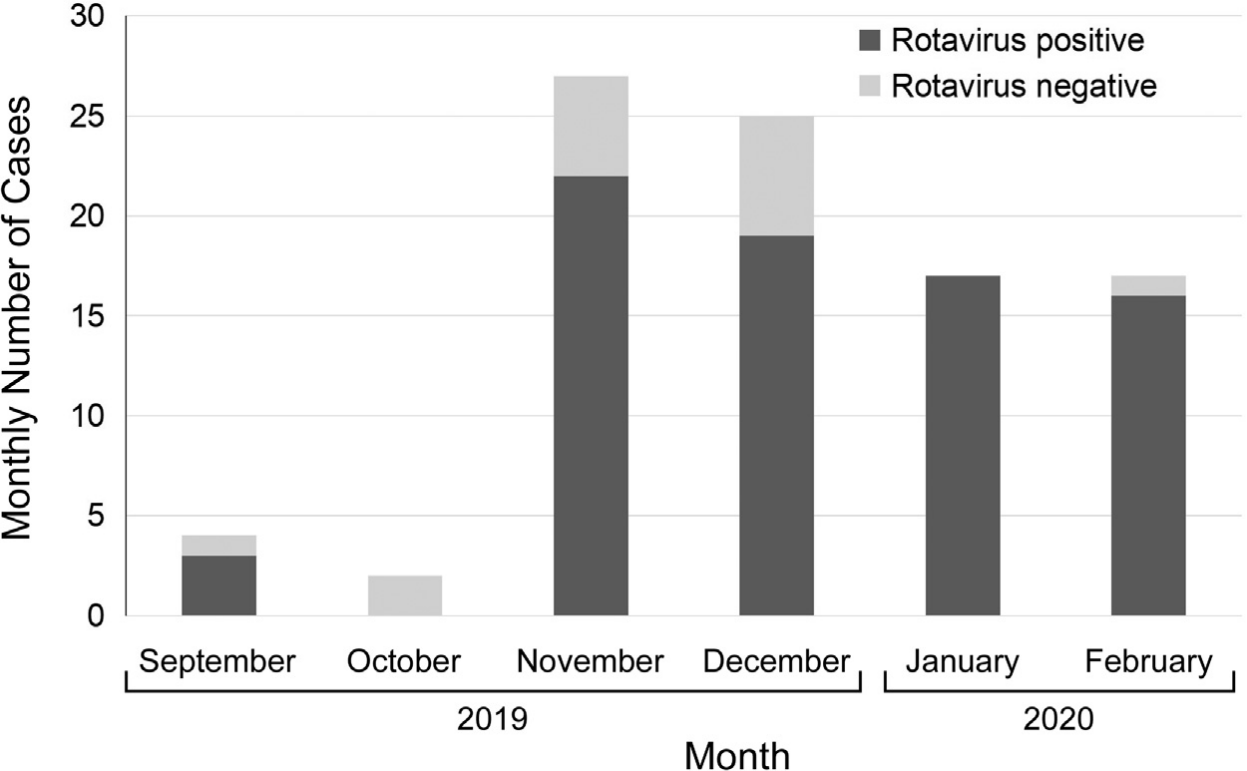
Monthly numbers of stool samples positive and negative for rotavirus. Rotavirus was detected in 75-100% of stool samples every month, except in October 2019.

Rotavirus was the most frequently detected pathogen by multiplex PCR (Figure 3). To determine the clinical impact of rotavirus infection on AGE severity, we compared the Vesikari scores between patients with positive and negative results for rotavirus using multiplex PCR. The median Vesikari scores for those with (10; IQR, 9-11) and without (11; IQR, 10-13) rotavirus infection did not differ significantly (*P* = 0.05).

### AGE severity in patients infected with one or multiple microorganisms

Next, we compared the severity of AGEs in patients infected with one or multiple microorganisms. The median Vesikari scores in patients with AGE infected with multiple microorganisms (11; IQR, 9-13) and one microorganism (12; IQR, 10-13) did not significantly differ (*P* = 0.10).

### Genotyping of rotavirus responsible for AGE

Of 77 rotavirus-positive stool samples, 73 were available for genotyping. Of these, 44 (60.3%) were successfully genotyped, while the remaining 29 (39.7%) were not. It was related that insufficient sample volume was left for genotyping in some samples, because approximately 500 μl of samples was used for the FilmArray^TM^ GI panel, and rapid antigen tests and amounts of sample collection were not consistent. Among the genotyped samples, the most common were G1P [8] (n = 19; 26.0%) and G2P [4] (n = 19; 26.0%), followed by G1P [4] (n = 3; 4.1%) and G1P [6] (n = 3; 4.1%) (Supplementary Figure).

## Discussion

In this study, the most common virus detected in hospitalized children with severe AGE was rotavirus, and the most common genotypes detected were G1P [8] and G2P [4]. Norovirus was the second most frequent viral pathogen, and EAEC was the most frequent bacterial pathogen.

In a systematic review of the causes of diarrhea worldwide in children younger than 5 years, EPEC and norovirus were frequently detected in stools after rotavirus infection. Cryptosporidium spp. and *Giardia lamblia* have been sporadically detected [14]. In the current study, 15 microorganisms other than rotaviruses were detected in the stool samples of pediatric patients with AGE in Yangon, Myanmar. In contrast to previous findings, EAEC and EPEC were detected more frequently than norovirus. Notably, parasites were not detected in the current study, which was not reported in a previous review [14]. The present study was conducted in Yangon, an urban area in Myanmar, and the patients mainly lived in the city and surrounding areas. The incidence of parasitic infections may differ between the non-urban and rural areas of Myanmar.

Rotavirus genotyping revealed that the most frequent rotavirus genotypes were G1P [8] (26.0%) and G2P [4] (26.0%). However, previous studies in Myanmar identified the dominant genotypes as G12P [8] (35.5%) and G1P [8] (15.3%), during 2009-2014, and G1P [6] (31.5%) and G1P [8] (26.2%), from 2018 to 2020 [6,7]. In previous reports, G2P [4] was not the dominant genotype (8.8% in 2009-2014 and 9.2% in 2018-2020). The present genotype distribution differed from that of previous reports, most likely because these epidemiological studies included a wide area of the country, including central and eastern Myanmar, and because the genotype distribution differed by season and year [6,7]. The monovalent rotavirus vaccine (RV1) was introduced in Myanmar in February 2020 [15]. RV1 was designed to target G1P [8] and also offers cross-protection against G3P [8], G9P [8], and G2P [4,16]. We assume that the COVID-19 pandemic in 2020 and the political disruption in 2021 adversely affected rotavirus vaccine distribution in Myanmar. If RV1 becomes widely distributed among Burmese children in the future, rotavirus genotype distribution could be affected by the vaccine, as previously reported [17,18].

Our study was conducted before the introduction of rotavirus vaccination and thus included data from the pre-rotavirus vaccination era. In some countries where rotavirus vaccination has begun, the number of hospitalizations for rotavirus AGE has declined [[19], [20], [21], [22]]. Therefore, the causative microorganisms of pediatric AGE, including non-rotavirus pathogens, are likely to change dramatically in these countries due to the widespread use of rotavirus vaccines. Although studies have compared the incidence of rotavirus infection before and after the start of rotavirus vaccination [23,24], few have comprehensively analyzed the causative microorganisms, including non-rotavirus pathogens, in relation to rotavirus vaccination status.

Other studies reported that rotavirus was the dominant pathogen in AGE in Myanmar and other countries, and rapid antigen tests detected rotavirus in almost half of the children with AGE [2,6,7]. In the present study of hospitalized patients with AGE, rotavirus was detected in 84% of the samples by multiplex PCR and in 50% of the samples by rapid antigen testing. Although the rotavirus positivity rate for rapid antigen testing was similar to that in previous reports from Myanmar (46-50%) [6,7], the multiplex PCR positivity rate (84%) was considerably higher. Thus, it is possible that rotavirus infection was more prevalent than past estimates suggest, as these estimates were based on the results of rapid antigen tests. Rotavirus can cause symptomatic or asymptomatic infections. In addition, rotavirus reinfection is common and usually results in less severe disease [25].

In previous studies, a lower sensitivity of the rapid antigen test for rotavirus was reported in patients with lower viral loads in stool samples by PCR assay [26,27]. In particular, the sensitivity of the rapid antigen test was observed to decrease in asymptomatic infections because stool samples of patients with asymptomatic rotavirus infection exhibited a lower viral load than those with symptomatic infection. Thus, multiplex PCR detection of rotaviruses may identify cases of asymptomatic or mild infection. Viral shedding of rotavirus in stool may continue for 1 month [27]. Because the sensitivity of the multiplex PCR assay is higher than that of rapid antigen testing, we were unable to determine whether the detection of rotavirus indicated a recent, ongoing, or asymptomatic infection. Quantitative PCR assays for rotavirus, as well as clinical signs and symptoms, may provide sufficient information to differentiate between these conditions.

We have demonstrated that rotavirus is the most important virus for children with AGE in Myanmar, and the rotavirus vaccine will definitely help prevent AGE caused by rotavirus in Myanmar. However, after the introduction of the rotavirus vaccine in 2020; the coup occurred in February 2021, which impacted the implementation of the vaccine significantly. As a result, it was reported that the vaccination coverage of EPI in Myanmar declined by 50% [5]. Thus, it is estimated that the control of VPDs, including rotavirus infection, has been unsuccessful in Myanmar, which threatens global public health. Further follow-up surveillance studies of pediatric AGE in Myanmar are needed. However, it has been difficult to perform clinical studies in general because of current political issues.

This study has several limitations that warrant mention. First, it focused on inpatients with moderate-to-severe AGE; however, the pathogen profile for AGE may have been different if our analysis included outpatients. Second, the study period was limited to 6 months. As previous perennial studies in Myanmar showed that pediatric AGE case numbers increased during the dry season [6,7], our study included the season in which AGE was most frequent in Myanmar. Therefore, a longitudinal study of AGE is necessary to monitor the continuous changes in microorganisms causing AGE in children in Myanmar. Third, we were not able to show the number of excluded cases due to limited human resources in the institution; however, the positive rate of rotavirus by rapid antigen test in the current study was similar to that in previous reports, and it is estimated that our study was able to capture similar rates of rotavirus infection in children in Myanmar (50% vs 46-50%) [6,7]. Finally, the sample size was small, because the study duration was limited by the need to complete the study before introducing the rotavirus vaccine.

In conclusion, this comprehensive epidemiological study, which was conducted before the introduction of rotavirus vaccination, showed that rotavirus was the predominant pathogen and was frequently detected along with other pathogens in stool samples from hospitalized children with AGE in Yangon, Myanmar. Because the AGE pathogen profile is likely to change dramatically after the introduction of the rotavirus vaccine, future studies should use the same assay to evaluate pediatric AGE during the rotavirus vaccination era.

## Declarations of competing interest

The authors declare that they have no known competing financial interests or personal relationships that could have appeared to influence the work reported in this paper.

## Appendix Supplementary materials

Supplementary Figure. Genotype distribution of rotavirus in Yangon, Myanmar. In total, 44 samples (60.3%) were successfully genotyped. Among the genotyped samples, the most common were G1P [8] (n = 19, 26.0%) and G2P [4] (n = 19, 26.0%). Image, application 1

## Footnotes

Supplementary material associated with this article can be found, in the online version, at doi:10.1016/j.ijregi.2025.100589.
