# Supplementary material for: Burden of rotavirus and other microorganisms in hospitalized children with acute gastroenteritis in Yangon, Myanmar, before the introduction of rotavirus vaccine
Source: IJID Reg. 2025 Jan 31;14:100589. doi: 10.1016/j.ijregi.2025.100589 (PMC11891738; doi:10.1016/j.ijregi.2025.100589)
Supplement: Supplementary file 1 — Supplementary Figure. Genotype distribution of rotavirus in Yangon, Myanmar. In total, 44 samples (60.3%) were successfully genotyped. Among the genotyped samples, the most common were G1P [8] (n = 19, 26.0%) and G2P [4] (n = 19, 26.0%). [file mmc1.pdf]

Supplementary Figure. Genotype Distribution of Rotavirus in Yangon, Myanmar

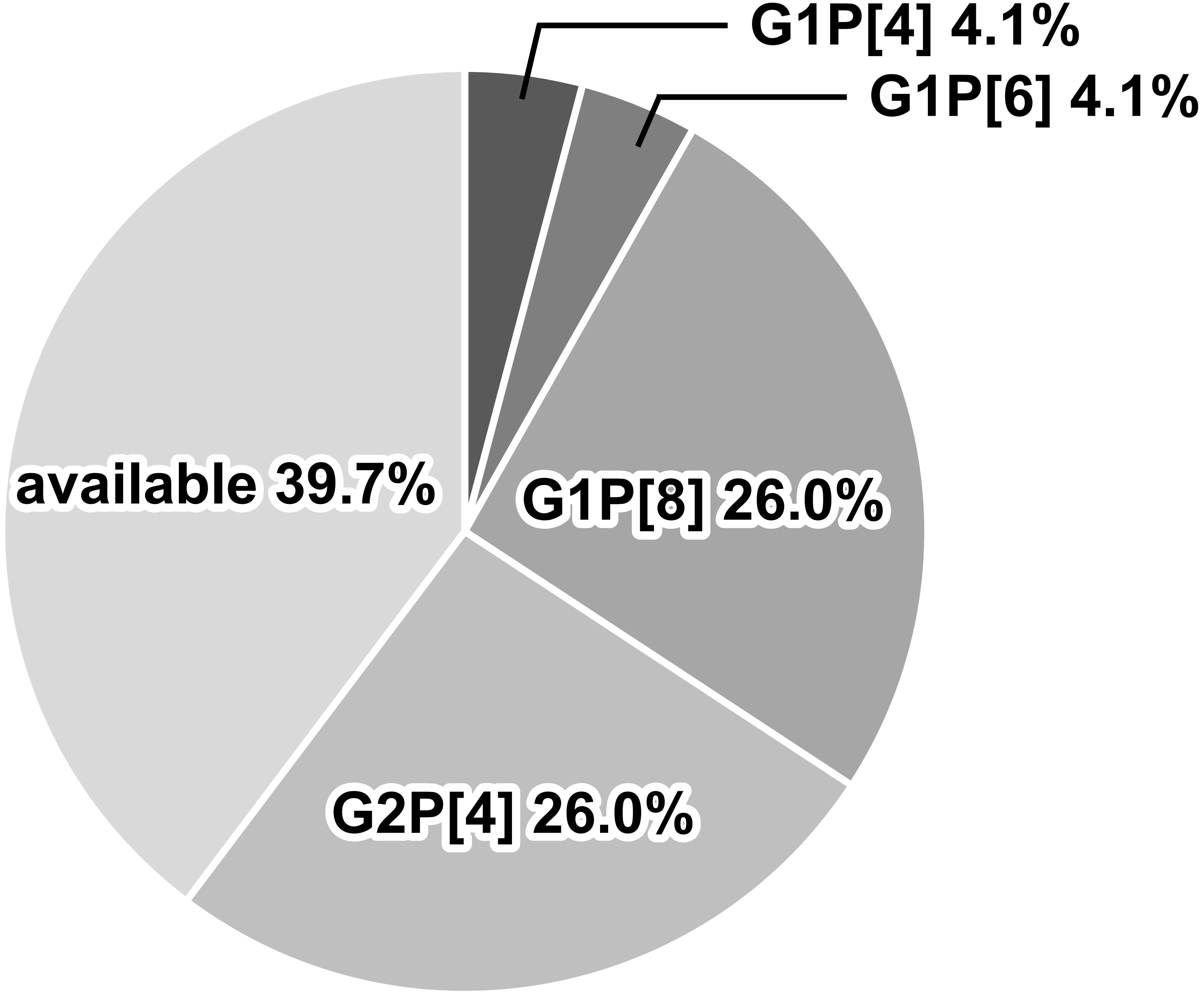

Supplementary Figure. Genotype Distribution of Rotavirus in Yangon, Myanmar

In total, 44 samples (60.3%) were successfully genotyped. Among the genotyped samples, the most common genotypes were G1P[8] (n = 19; 26.0%) and G2P[4] (n = 19; 26.0%).
